# Management of Symblepharon Prior to Keratoprosthesis in Chronic Ocular Burns: A Sequential Approach

**DOI:** 10.7759/cureus.24611

**Published:** 2022-04-29

**Authors:** Anahita Kate, Mariya B Doctor, Swapna S Shanbhag

**Affiliations:** 1 Cornea and Anterior Segment, L V Prasad Eye Institute, Vijayawada, IND; 2 Cornea and Anterior Segment, L V Prasad Eye Institute, Hyderabad, IND

**Keywords:** amniotic membrane transplant, limbal stem cell deficiency, mucous membrane graft, symblepharon, keratoprosthesis, ocular chemical burns

## Abstract

This report describes two cases with stepwise management of chronic ocular burn sequelae with concurrent total limbal stem cell deficiency (LSCD) and advanced symblepharon. Both were mono-ocular patients with the other eye being phthisical. In both cases, a sequential approach was planned, and at the first stage, the symblepharon was released to stabilize the ocular surface and form the fornices. This was followed by a type 1 keratoprosthesis (KPro) after four months of symblepharon release in the first case, and after nine months in the second case. In the first case, after symblepharon release, the resultant bare sclera was addressed with an oral mucous membrane graft (MMG). Over two years of follow-up, there was no evidence of recurrence of the symblepharon. No further surgical interventions were required. In the second case, after symblepharon release, the resultant bare sclera was addressed with a cryopreserved amniotic membrane (AM). Over eight years of follow-up, six episodes of recurrence of the symblepharon were noted over the optic of the KPro, necessitating trimming of the conjunctiva from over the optic. Thus, with these two cases, we would like to emphasize that addressing adnexal pathologies such as a symblepharon with an oral MMG before implanting a KPro, may help prevent further recurrences of symblephara and the need for multiple surgical interventions. The oral mucosa is a better alternative to the conjunctiva as compared to the AM in a mono-ocular patient where conjunctiva cannot be harvested from the contralateral eye.

## Introduction

Various chronic sequelae ensue from ocular chemical burns of which limbal stem cell deficiency (LSCD) is perhaps one of the most visually debilitating. In a study that described the etiologies of LSCD, ocular chemical burns were the most common cause for both unilateral and bilateral LSCD [1]. The management of such eyes is based on the ocular surface wettability, the severity of LSCD, and the fellow eye status; with a limbal stem cell transplant (LSCT) being the preferred modality of therapy. However, because of the presence of significant adnexal damage and corneal scarring, LSCT may not be feasible in all cases or may yield suboptimal outcomes unless combined with ocular surface reconstruction or a keratoplasty. In bilateral LSCD with no autologous source of limbal epithelial stem cells (LESCs), immunosuppression is warranted as an allogeneic source of the donor is used. A keratoprosthesis (Kpro) circumvents these issues by providing a one-step procedure with faster visual rehabilitation in these eyes without the need for systemic immunosuppression [2]. For protecting the cornea from evaporative and erosive damage post-KPro implantation, a large diameter contact lens is placed and changed every three months [3]. When symblephara with attachments to the cornea are not addressed adequately before KPro implantation, the fibrotic tissue may progress to cover the optical cylinder and obscure the visual axis. In the current report, we present two cases where the symblepharon was addressed before implanting the KPro. However, the tissues that were used as a substitute to the conjunctiva post symblepharon release were different, thus leading to different outcomes, complications, and re-surgery rates.

## Case presentation

Case 1

**Figure 1:**
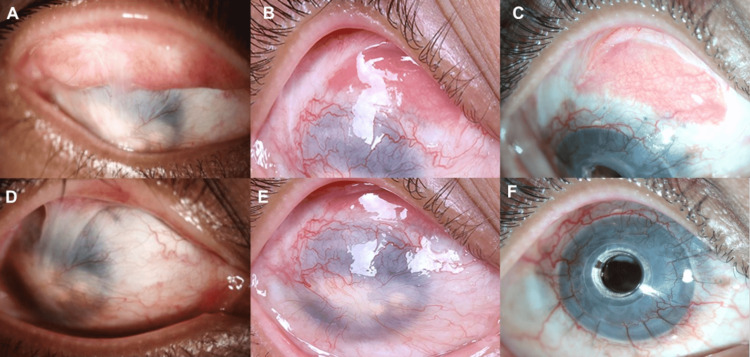
Sequential approach for an eye with total limbal stem cell deficiency (LSCD) with symblepharon for which, the symblepharon release was done with a mucous membrane graft (MMG) followed by type 1 keratoprosthesis (KPro) implantation. A: Pre-operative image of the everted right upper lid depicting the extension of the superior symblepharon on the tarsal area; B: At four months postoperatively after the oral mucous membrane graft (MMG) surgery, a well-integrated MMG is noted superiorly; C: At 28 months postoperatively after KPro surgery, the MMG is stable with no recurrence of symblepharon; D: Pre-operative image of the cornea, and total LSCD with central leucomatous scarring; E: At four months postoperatively after MMG, no recurrence of the symblepharon was seen over the cornea; F: At 28 months postoperatively after KPro surgery, the ocular surface is stable. KPro: Type 1 keratoprosthesis, MMG: Mucous membrane graft, LSCD: Limbal stem cell deficiency

A 26-year-old male presented with a history of fall of lime in both eyes 12 years prior to presentation. He had undergone a penetrating keratoplasty and a KPro in the left eye. A retinal detachment was observed one month following the KPro implantation and the eye subsequently became phthisical. At presentation, he had a visual acuity of perception of light with an accurate projection of rays in the right eye. There was no perception of light in the left eye. In the right eye, the lid margins were normal, and the ocular surface was wet with a Schirmer’s value of 9 mm after five minutes. A superior symblepharon was observed extending 4 mm onto the corneal surface. Total LSCD with central leucomatous scar measuring 6 mm x 8 mm was present with retained lime foreign bodies (Figure 1 A and D). On B-scan ultrasonography of the right eye, an anechoic vitreous cavity with an attached retina was noted. The patient underwent a diagnostic endoscopy to assess the posterior segment structures in the right eye which revealed a normal fundus with a healthy macula. A diagnosis of right eye chronic ocular burn sequelae with total LSCD with advanced superior symblepharon was made. A type 1 KPro was planned in the right eye. However, since the placement of a large diameter contact lens would be required post-KPro implantation, it was decided to first address the symblepharon with an oral labial mucous membrane graft (MMG) followed by KPro for visual rehabilitation. 

The surgery was performed under general anesthesia. Adequate surgical exposure was obtained by passing two stay sutures in the upper lid with 4-0 silk. The symblepharon was separated from the corneal surface with blunt dissection and this was continued over the superior conjunctival area. The underlying Tenon's layer was also dissected and a 12 mm x 6 mm bare scleral area was created. A labial mucosal graft measuring 13 mm x 7 mm was harvested from the lower lip after creating a submucosal bleb with a solution of 2% lignocaine and adrenaline (1:200 000). The edges of the donor area were sutured with four interrupted 6-0 polyglactin sutures for faster healing. The graft was trimmed to reduce the submucosal adipose tissue and the minor salivary glands and sutured onto the superior bare sclera with interrupted 7-0 polyglactin suture and fibrin glue (TISSEEL, Baxter AG, Vienna, Austria). The superior de-epithelised corneal surface from where the symblepharon was released was covered with cryopreserved AM. A bandage contact lens (BCL) was placed upon completion of the procedure.

Postoperatively, the MMG was in situ and the ocular movements were full. The patient was started on topical antibiotics (moxifloxacin 0.5%, four times/day), corticosteroids (prednisolone acetate 1%, eight times/day) and lubricants (carboxymethylcellulose 0.5%, 12 times/day). An anesthetic lip gel (choline salicylate) application before meals was advised along with chlorhexidine mouth wash after meals. The healing of the donor area was complete without any complications at two weeks. The topical steroids were gradually tapered over six weeks. At four months following MMG surgery, there was no recurrence of symblepharon, and a stable ocular surface was noted (as seen above in Figure 1 B and E). The patient subsequently underwent an AuroKPro (Aurolab, Madurai, India) performed using a standardized technique [4]. A large diameter contact lens of 16 mm diameter (SilkLens, Bengaluru, Karnataka, India) was placed on the cornea at the end of the surgery. The visual acuity improved to 20/80 within three weeks of the surgery and the patient was continued on topical antibiotics (moxifloxacin 0.5%, four times/day) and steroids (prednisolone acetate 1%, four times/day). The patient underwent a replacement of the contact lens every three months. At the last follow-up visit two years after the KPro surgery, the patient had a well epithelized ocular surface with a stable KPro and no recurrence of the symblepharon (as seen above in Figure 1 C and F). The best-corrected visual acuity (BCVA) was 20/40 and the posterior segment was normal with a healthy disc. The patient was maintained on moxifloxacin 0.5% (two times/day) and prednisolone acetate 1% (once daily).

Case 2

**Figure 2:**
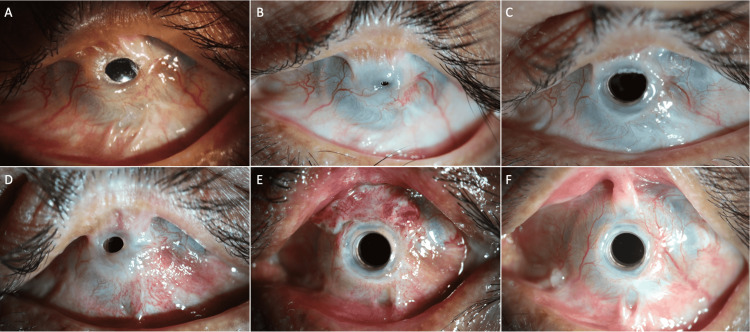
Sequential approach for an eye with total limbal stem cell deficiency with symblepharon for which, a symblepharon release was performed with amniotic membrane (AM) graft followed by Type 1 keratoprosthesis (KPro) implantation. A: Post symblepharon release with Type 1 KPro implantation, recurrence of symblepharon observed superiorly; B: Recurrence of symblepharon after three years which covered the entire optic of the KPro; C: Trimming of the conjunctiva over the optic of the cylinder was performed and improvement in visual acuity was seen; D: Recurrence of symblepharon noted over the optic six years after KPro implantation; E: Symblepharon release with AMG was performed for one such recurrence; F: The status of the eye at the last postoperative follow-up visit at 8.5 years after KPro implantation reveals early recurrence of symblepharon superiorly

A 39-year-old male presented with a history of acid injury in both eyes four months prior to presentation. The right eye was phthisical. The left eye had a visual acuity of perception of light with an accurate projection of rays, the lid margins were normal and a wet ocular surface was noted. Total LSCD with superior advanced symblepharon encroaching onto the cornea was seen. On B-scan ultrasonography, an anechoic vitreous cavity with an attached retina was noted. A diagnosis of left eye chronic ocular burn sequelae with total LSCD with advanced superior symblepharon was made. Symblepharon release was performed superiorly, and cryopreserved AM was placed on the resultant bare sclera. Nine months after surgery, a recurrence of the symblepharon was noted. A Type-1 KPro was performed and repeat symblepharon release with AM grafting was performed simultaneously. The BCVA was maintained at 20/30 (Figure 2 A). However, three years after the KPro surgery, the BCVA dropped to 20/320 with recurrence of the symblepharon with conjunctiva encroaching onto the central optic of the KPro, thus obscuring the visual axis (Figure 2 B). Trimming of the conjunctiva was performed clearing the central optic (Figure 2 C). The BCVA again improved to 20/30. After this recurrence, five more episodes of such recurrences of symblepharon and occlusion of the optic of the Kpro were noted (Figure 2 D). Overall, post the first symblepharon release, seven episodes of recurrence were noted. Trimming of the conjunctiva from over the cylinder was performed in the operating theatre at each visit for five such episodes. For one episode of recurrence, symblepharon release with AM graft was performed (Figure 2 E). Overall, post-KPro surgery, the patient underwent six such surgeries. The last recurrence was noted eight years post-KPro implantation. Gradual increase in the symblepharon with slow encroachment of the conjunctiva over the optic is noted after each surgery (Figure 2 F). The patient has been advised to undergo symblepharon release with mucous membrane graft at multiple visits but has not yet given consent for the same. Due to multiple recurrences of the symblepharon, retention of a large diameter contact lens has not been possible in the last three years. However, BCVA is maintained at 20/30 after trimming the conjunctiva over the optic.

## Discussion

In eyes with a wet ocular surface, total LSCD, and symblepharon, the management options include either an ocular surface reconstruction with LSCT or a KPro. The rapid visual recovery and the single-sitting technique make KPro an attractive option in such cases. Symblepharon following ocular burns is fairly common and can occur in up to 18% of cases with severe surface burns [5]. The presence of a symblepharon by itself is not an indication of surgical intervention. This is typically reserved for cases where the cicatricial tissue hampers the placement of a contact lens, restricts ocular mobility, or causes significant ptosis or lagophthalmos. Correction of a symblepharon prior to KPro implantation may not be necessary for eyes where the symblepharon is peripheral and not abutting the cornea. However, if the symblepharon is encroaching onto the cornea, it is prudent to address this appropriately prior to KPro surgery to prevent recurrences and subsequent surgeries. 

Several options are available to cover the resultant bare area over the sclera after the excision of a symblepharon. These include an AM, conjunctival autograft (CAG), or an oral MMG. Solomon et al. reported the outcomes of AM transplantation (AMT) after symblepharon lysis to form the conjunctival fornices, specific to eyes with ocular chemical burns [6]. In their study, three eyes with ocular chemical burns had successful restoration of the fornices with AMT over a follow-up period of 15 to 72 months. However, the grading of symblepharon in these eyes pre-operatively is unclear. If the symblepharon is smaller in size and limited to the fornices, then the growth of the conjunctival epithelium over the AM can reduce recurrences. However, in advanced cases of cicatrization, with symblepharon occupying a larger area, there may not be an adequate number of residual conjunctival epithelial cells present to migrate over the AM. In such cases, an increased risk of recurrence of cicatrization has been associated with the use of an AM, which was also seen in the second case [7]. Younger age, underlying autoimmune diseases, and a history of prior ocular surgeries are also associated with a higher risk of recurrence [6,7]. In another study by Tseng et al., intra-operative use of mitomycin-C in conjunction with AMT for fornix reconstruction in severe cicatricial ocular surface disorders post ocular chemical burns in seven eyes were found to be successful in six eyes over a follow-up period of nine to 19 months [8]. Although no side effects were noted in their study secondary to the use of mitomycin-C, it is still prudent to keep such patients on a close follow-up. Such an approach was avoided in our case as the symblepharon covered one-third of the cornea and in such a scenario, the use of mitomycin-c on the bare sclera or underneath the residual conjunctiva could have delayed epithelial healing in the area of the cornea from where the symblepharon was released. In a study by Jain et al., symblepharon release with AMT was performed in eight eyes with ocular chemical burns and a successful outcome was noted in six eyes over a follow-up period of five to 25 months [9]. In the two eyes with recurrence, failure was attributed to severe dry eye and previous conjunctival surgery. An upregulation in the transforming growth factor-β signaling can promote a subconjunctival fibroblastic reaction which may trigger the recurrent episodes [7].

Conjunctival autografts in conjunction with AMT have been used to address symblepharon with excellent success rates [10,11,12]. A conjunctival autograft from the ipsilateral or the contralateral eye is an excellent option to cover the bare sclera in the eyes after symblepharon release since this is direct transplantation of an epithelial layer with the same phenotype. However, using a conjunctival autograft was not an option in our case secondary to the bilateral pathology. Also, in eyes with advanced cicatrization, there may not be sufficient healthy conjunctival tissue to harvest from the ipsilateral or the contralateral eye, as was the case in our patient.

The outcomes of oral MMG for symblepharon secondary to ocular chemical burns have been encouraging. In a study by Martinez-Osorio et al., six eyes with ocular chemical burns underwent symblepharon release with labial mucosal grafts and none of the eyes had a recurrence with a follow-up period of 36 to 44 months [13]. In eyes with concomitant dry eye, the submucosal glands can also be retained to improve the wetness of the ocular surface [14,15]. Kheirkhah et al. provided a grading system where symblephara were divided into four grades based on their severity, and cicatrix lysis with AMT showed good success rates in smaller symblephara while anchoring sutures, intra-operative mitomycin-c, conjunctival autografts, and oral mucosal grafts were required for more advanced symblephara [16]. Another prospective study by Kheirkhah et al. also showed a success rate in 85% of eyes with advanced symblephara with the combined approach of cicatrix lysis, mitomycin-c application, MMG, and sutureless AMT [17]. However, in both studies by Kheirkhah et al., the oral mucosal graft was sutured to the tarsus and not to the palpebral surface, as was performed in our case, and the AM was secured to the bare sclera. In our case, MMG was sutured to the bare sclera and no tissue was required over the tarsus after the symblepharon release.

Prior to KPro implantation, it is essential to address the symblepharon appropriately as a recurrence can encroach onto the optic of the KPro and substantially affect the visual function. Patients requiring a KPro usually have bilateral visual dysfunction and hence, the resultant visual morbidity significantly affects the quality of life in such individuals. Also, if the symblepharon is not addressed prior to performing a KPro, fitting the eyes with a large diameter contact lens post-KPro may not be possible, which leaves the cornea exposed and at the risk of desiccation. Furthermore, the multiple surgeries performed may cause repeated disruptions of the tear film and increased surface inflammation.

## Conclusions

The current case emphasizes the need for addressing symblephara pre-emptively in eyes that require a KPro. A meticulous preoperative examination will help detect the eyes that will require symblepharon release prior to surgical interventions for visual rehabilitation. In mono-ocular patients where a conjunctival autograft cannot be harvested, oral MMG sutured to the bare sclera after symblepharon release can lead to a successful long-term anatomical outcome without recurrences and further interventions.
